# COVID-19 coronavirus: recommended personal protective equipment for the orthopaedic and trauma surgeon

**DOI:** 10.1007/s00167-020-06022-4

**Published:** 2020-04-27

**Authors:** Michael T. Hirschmann, Alister Hart, Johann Henckel, Patrick Sadoghi, Romain Seil, Caroline Mouton

**Affiliations:** 1 Department of Orthopaedic Surgery and Traumatology, Kantonsspital Baselland (BruderholzLiestalLaufen), 4101 Bruderholz, Switzerland; 2 University of Basel, 4051 Basel, Switzerland; 3 UCL Stanmore Campus, Royal National Orthopaedic Hospital Brockley Hill, Stanmore, UK; 4 Department of Orthopedics and Trauma, Medical University of Graz, Auenbruggerplatz 5, 8036 Graz, Austria; 5 Department of Orthopaedic Surgery, Centre Hospitalier de Luxembourg-Clinique d’Eich, 78 Rue d’Eich, 1460 Luxembourg, Luxembourg; 6 Luxembourg Institute of Health, 78 Rue d’Eich, 1460 Luxembourg, Luxembourg

**Keywords:** COVID-19, Corona, Surgeon, Protection, Personal protective equipment, Masks, Suits, Helmet, Respirator masks, Aerosols

## Abstract

**Purpose:**

With the COVID-19 crisis, recommendations for personal protective equipment (PPE) are necessary for protection in orthopaedics and traumatology. The primary purpose of this study is to review and present current evidence and recommendations for personal protective equipment and safety recommendations for orthopaedic surgeons and trauma surgeons.

**Methods:**

A systematic review of the available literature was performed using the keyword terms “COVID-19”, “Coronavirus”, “surgeon”, “health-care workers”, “protection”, “masks”, “gloves”, “gowns”, “helmets”, and “aerosol” in several combinations. The following databases were assessed: Pubmed, Cochrane Reviews, Google Scholar. Due to the paucity of available data, it was decided to present it in a narrative manner. In addition, participating doctors were asked to provide their guidelines for PPE in their countries (Austria, Luxembourg, Switzerland, Germany, UK) for consideration in the presented practice recommendations.

**Results:**

World Health Organization guidance for respiratory aerosol-generating procedures (AGPs) such as intubation in a COVID19 environment was clear and included the use of an FFP3 (filtering face piece level 3) mask and face protection. However, the recommendation for surgical AGPs, such as the use of high-speed power tools in the operating theatre, was not clear until the UK Public Health England (PHE) guidance of 27 March 2020. This guidance included FFP3 masks and face protection, which UK surgeons quickly adopted. The recommended PPE for orthopaedic surgeons, working in a COVID19 environment, should consist of level 4 surgical gowns, face shields or goggles, double gloves, FFP2-3 or N95-99 respirator masks. An alternative to the mask, face shield and goggles is a powered air-purifying respirator, particularly if the surgeons fail the mask fit test or are required to undertake a long procedure. However, there is a high cost and limited availabilty of these devices at present. Currently available surgical helmets and toga systems may not be the solution due to a permeable top for air intake. During the current COVID-19 crisis, it appeared that telemedicine can be considered as an electronic personal protective equipment by reducing the number of physical contacts and risk contamination.

**Conclusion:**

Orthopaedic and trauma surgery using power tools, pulsatile lavage and electrocautery are surgical aerosol-generating procedures and all body fluids contain virus particles. Raising awareness of these issues will help avoid occupational transmission of COVID-19 to the surgical team by aerosolization of blood or other body fluids and hence adequate PPE should be available and used during orthopaedic surgery. In addition, efforts have to be made to improve the current evidence in this regard.

**Level of evidence:**

IV.

## Introduction

COVID-19 coronavirus has spread dramatically over the entire globe affecting all health-care systems [29, 30]. In most countries around Europe, a discussion has started on how to optimally protect health-care workers [12–14, 16]. There is a variety of different recommendations for health-care worker protection given by each country or hospital. However, there are to date no clear recommendations for personal protective equipment (PPE) and safety recommendations in the surgical field such as orthopaedics and traumatology.

The recommendations should lead to an optimal protection and safety of all health-care workers. A COVID-19 infection of just one health-care worker can have a dramatic effect for the health care itself. It is known that the average person infected with COVID-19 is likely to infect 1.5 to 3.5 others [4]. A single COVID-19 infection among essential health-care workers at a hospital might severely reduce the capacity of an entire hospital [14]. In Wuhan, the outbreak region, around 1300 health-care workers became infected [24]. In Italy to date, over 100 physicians have died of the disease [14]. The likelihood of becoming infected for health-care workers is more than three times as high as the general population.

There is general consensus that all health-care workers should wear regular surgical masks and gloves for all patient interactions. In addition, avoiding unnecessary contact, keeping adequate distance, proper hand hygiene and disinfection is indicated. In most hospitals, positive or suspicious for COVID-19 patients are separated from non-infected patients. Handling of COVID-19-negative patients is difficult, because many COVID-19 patients are asymptomatic, the availability of screening tests is often limited and they have a high rate of false-negative findings [23].

There is an uncertainty regarding the optimal PPE for different tasks in our daily work. There is general agreement that respiratory masks should be used for aerosol-generating procedures (AGPs). However, there has been confusion regarding the definition of AGPs in orthopaedic surgery and traumatology. In fact, recommendations for PPE have been influenced by the availability of adequate masks, gloves, gowns, helmets and goggles rather than the science for their use.

Although elective surgery might have been postponed in some countries, trauma and orthopaedics is at the frontline with coronavirus, because emergency orthopaedic operations are still amongst the most common emergency surgical procedures. Many orthopaedic surgeons do not know what is safe to wear and recommended. With increasing numbers of fatalities among doctors, we aimed to raise awareness of the issues surrounding PPE by reviewing current evidence and recommendations for PPE in orthopaedic surgery and traumatology.

## Material and methods

A systematic review of the available literature was performed using the keyword terms “COVID-19”, “Coronavirus”, “surgeon”, “health-care workers”, “protection”, “masks”, “gloves”, “gowns”; “helmets”, “aerosol”, “telemedicine” in several combinations. The following databases were assessed: Pubmed (https://www.ncbi.nlm.nih.gov/sites/entrez/), Cochrane Reviews (https://www.cochrane.org/reviews/), Google Scholar (https://scholar.google.com/).

All the publications from 01.01.2004 to 01.04.2020 were searched. The search was limited to English and German studies only. Studies in other languages were not included in this review. In addition, articles were obtained from other references, WHO or via Google Search.

All peer-reviewed articles were considered. Randomized controlled trials (RCTs), prospective trials and retrospective studies as well as reviews and case reports were included in this systematic review. Two authors independently screened the titles and abstracts of all the articles identified. If the abstract was unavailable, the paper was excluded. In the event of disagreement, a consensus was reached by discussion, if needed with the intervention of the senior author.

This systematic review was conducted in accordance with the established guidelines from Preferred Reporting Items for Systematic Reviews and Meta-analysis (PRISMA).

However, due to the heterogeneity of available data it was decided to present the review in a narrative manner.

### Data extraction

One author extracted data from all the selected original articles, which was repeated by two other authors. If there was no agreement between the three, the senior author was consulted. Where required, the corresponding authors were contacted for additional information. Data were extracted from each included article and entered into a spreadsheet for analysis. Pertinent information extracted included author, date and journal of publication, study design (and level of evidence), and patient demographics (mean age, mean follow-up, total and group’s number of patients, outcome).

## Results

### Aerosol-generating procedures (AGPs)

Four studies were found which deal with the question of aerosol generation during surgery.

In the landmark article by Nogler et al., which was published in 2001, the authors performed three laminectomies (C4–C6) in cadavers using a high-speed 0.6-mm ball cutter [25]. They investigated the environmental and body contamination through contaminated aerosols generated by this high-speed cutter. The irrigation solution was marked with *Staphylococcusaureus* and after surgery used for tracing of aerosol contamination. The authors found a contamination at an area of 5 × 7 m around the operating field and everyone in the room showed face and body contamination. The surgeon and the surgical assistant showed a more severe contamination than other OR staff [25].

In another cadaver study with a similar setup by Nogler et al., the authors investigated environmental and body contamination by an ultrasound device and a high-speed cutter in the revision of cemented total hip arthroplasty [26]. The authors found an environmental contamination of 6 × 8 m. Both the ultrasound and the high-speed cutter contaminated all members of the surgical team consisting of an anaesthesiologist, a surgeon, a surgical assistant and a scrub nurse [26].

Heinsohn et al. investigated the exposure of operating room (OR) personnel to blood aerosols and found that the upper respiratory tract was exposed to aerosolized blood in the operating room [15].

Jewett et al. assessed whether different surgical power tools such as bone saws and bone drills or electrocautery in cutting and coagulation mode lead to aerosol generation in an OR setting [18]. All of the tools tested led to blood-containing aerosol particles < 5 µm.

Aerosol-generating procedures should be defined as respiratory or surgical. Respiratory AGPs, such as intubation, are a high risk of transmitting respiratory virus infections, such as COVID-19. Surgical AGPs, such as the use of high-speed power tools, are a high risk of transmitting virus particles in body fluids and pieces of body tissue; COVID-19 is known to be present in all body fluids.

### Personal protective equipment (PPE)

A narrative review by Wong et al. highlighted the fact that based on current evidence, aerosols might be generated during use of high-speed orthopaedic power tools [34]. They concluded that every person present during surgery should wear PPE including surgical gloves, a water-resistant surgical gown with long sleeves, a surgical mask, and full-face protection with a face shield [34]. In cases of possible airborne transmitted diseases (such as COVID-19), additional respiratory PPE should be used. In addition, they recommended to avoid use of electrocautery and power tools and to use wound irrigation with bulb syringes instead of pulsed irrigation (jet lavage) [34].

### Gowns protect skin and clothing

Sterile surgical gowns are part of the standard protection in the OR. In every surgery, the OR team consisting of the surgeon, the surgical assistants and the scrub nurse wear sterile surgical gowns to reduce intraoperative wound contamination and to minimize the patients` infection risk. It is also a personal protection against blood and body fluids, which are often sprayed in an area of 3–8 m around the operating table [25, 26].

Different types of surgical gowns offer different degrees of barrier protection to surgeons. The American Society for Testing and Materials (ASTM) F2407 is an umbrella document, which describes testing for surgical gowns: tear resistance, seam strength, lint generation, evaporative resistance, and water vapour transmission [3].

The critical zone of a surgical gown comprises the front area of the gown from chest to knees and the sleeves from the cuff to above the elbow. The safety levels of gowns for medical use can be classified in levels 1–4 [3]. Level 1 gowns should be used in minimal risk environment such as basic care or for visitors [3]. Level 2 gowns should be used in low-risk procedures such as venous blood draw [3]. Level 3 gowns are generally used for moderate-risk procedures such as arterial blood draw, or in the ER [3]. Level 4 gowns are preserved for high-risk procedures such as surgery or when infectious diseases are suspected [3].

Helmets or togas might also be an option for protection against body spray, but only protect against airborne transmission of COVID-19 in combination with respirator masks.

### Face masks protect mouth and nose

Generally, there are three different types of disposable masks available: single-use face masks, surgical masks, and respiratory masks.

Single-use face masks, which are typically thin and consist of only one layer, are only capable of filtering rather larger particles (3 μm). Surgical masks are generally more effective than single-use face masks in filtering virus-sized particles. A medical or surgical mask may be sufficient to prevent droplet transfer, while a respirator mask is required for airborne infection. However, the exact filtration characteristics of surgical masks are rather variable and depend on the layers used.

Most of the health-care workers currently use surgical masks to protect themselves against pathogens spread by droplet transmission such as COVID-19. Although it is well established that these provide insufficient protection against airborne transmission, there is conflicting evidence from a systematic review by Leung et al., which found that surgical masks can efficaciously reduce coronavirus detection and viral copies in large respiratory droplets and in aerosols [21]. However, this only suggests that it could be used by COVID-19-positive patients to limit further COVID-19 transmission. Clearly, this study has no relevance for OR staff protection [21].

The general consensus among surgeons is that conventional surgical masks do not offer protection against high-risk AGPs. Multiple surgical masks also fail to filter virus loaded particles. They should not be used as a substitute for respirator masks unless there is no alternative and the compromise for a lower level protection is made due to lack of availability [2, 13, 14].

For protection against airborne transmission, air-purifying respirator masks should be used. Respirator masks generally filter more smaller sized particles (0.3 μm) than surgical masks. The European Standard (EN 149:2001) classifies respirator masks into three different categories: filtering facepiece 1 (FFP1), FFP2, and FFP3. FFP2 is comparable to US standard N95 [20]. The filtration effectiveness of different masks is presented in Table 3.

In comparison to surgical masks, respirator masks show protection factors 11.5–15.9 times greater than those of surgical masks [20]. In addition to the type of masks used, the fitting and sizing of the mask is of utmost importance. Only a perfect-sized and well-fitted mask leads to efficient sealing of the respiratory tract. Intact masks can be worn for up to 8 h continuously [20].

Powered air-purifying respirators [11] were mainly used during the SARS outbreak for health-care personnel involved in high-risk invasive procedures or AGPs. These respirators in the form of a hood or a full-face mask consist of a motor-driven fan guiding the possibly contaminated air towards a filter, which then actively filters it and finally delivers the clean air to the user's face and/or mouth.

A recent systematic review of four randomised controlled trials by Bartoszko et al. compared medical masks to N95 respirator masks in their efficacy to prevent coronavirus in health-care workers [7]. The authors found and concluded that low certainty evidence exists that medical masks and N95 respirators offer similar protection against viral respiratory infection including coronavirus in health-care workers during non-aerosol-generating care [7].

### Eye protection using googles or face shields

Eye protection is critical for orthopaedic surgeons, as many procedures such as the use of power tools frequently lead to contamination of every OR personnel in the room and surface contamination in the OR in an area of up to 6 maround the operating table [25, 26].

In addition, a splash injury to the eye region, although less frequent than in the mouth and nose region, is a common event in surgeries such as total hip and knee arthroplasties, mainly affecting the operating surgeon and the assistant [1]. Therefore, orthopaedic surgeons must protect themselves from conjunctival contamination.

In a prospective study of conjunctival contamination during common orthopaedic operations, 43 (65%) of 66 goggles worn by surgeons were contaminated. The contamination rate of the protective flaps at the sides of the goggles was relatively low (5%), suggesting that ordinary spectacles, which are more convenient and comfortable than standard goggles, provide adequate protection during routine use [9, 10]. However, in an in vitro study aiming to compare the effectiveness of various types of protective eyewear in preventing conjunctival contamination during a femoral osteotomy, disposable plastic glasses were found to be associated with the lowest rate of conjunctival contamination (3%) and the authors recommended that eye protective devices should provide protection above and below the eye as well as contoured side protection to minimize the risk of contamination [22]. Modern prescription glasses provided no more benefit than the use of no eye protection, so that they should not be used as sole eye protection during surgical procedures.

Although there is no evidence to date, it is considered possible that SARS-CoV-2 is transmitted to the conjunctiva by aerosol. Preventive measures should thus include the systematic wear of goggles covering the eyes and the periocular skin for all health-care workers present in the room during potentially infectious aerosol-generating procedures.

### Gloves protect hands

Most patient care activities require the use of a single pair of nonsterile gloves made of either latex, nitrile, or vinyl. Sterile gloves are considered as standard protection in the OR, as they reduce the risk of exposure to blood-borne pathogens. For most orthopaedic surgeries, double gloving is recommended.

In orthopaedics and traumatology surgeries, surgical glove perforations have been reported to occur in 18.5% of conventional and 5.8% of arthroscopic procedures. They were more often seen in emergency surgeries compared to elective surgeries and mainly concerned the principal surgeon [19]. Also, more glove perforations occur during operations on bone compared with soft tissue operations [31].

The risk of contamination from blood is known to be 13 times higher when using single compared with double gloves, so that the use of double gloving is a recommended practice [19]. Exposure of surgeons to blood could indeed be reduced from 54 to 10%, by double gloving [31]. Finally, to reduce the risk of contamination and perforation [8], increasing the number of outer glove renewals during certain stages of total hip arthroplasty implantation such as prosthesis reduction, surgical incision, or femoral cementing is also recommended [31].

Limited data have shown that viral RNA could be detected in blood samples and it is not yet clear whether blood transmission of SARS-CoV-2 is possible. Preventive measures should include double gloving with outer glove renewals during at-risk procedures.

### Electronic personal protective equipment—telemedicine (ePEP)

Telemedicine has been recognized as an efficient tool for providing electronic personal protective equipment (ePPE) to health-care workers [32]. It might help to protect staff and save PPE while providing rapid access to emergency care in orthopaedics. Under certain circumstances, the service can be provided with limited direct physical patient contact. In most orthopaedic centres around the world, outpatient work had to be reduced or postponed due to COVID-19 crisis. Only patients with urgent orthopaedic conditions are seen face to face under PPE conditions. Consequently, video or telephone consultations are considered or done for many orthopaedic patients.

To date, there is only a paucity of studies dealing with the impact of telemedicine in orthopaedics. Prada et al. presented a tele-orthopaedic strategy and evaluated its efficiency and impact on waiting times for orthopaedic specialty consultations in a rural hospital in Chile [27]. The authors found that of 89 patients referred to the orthopaedic surgeon by telemedicine, 69.7% required one or more follow-ups through tele-orthopaedic service and 30.3% were referred for on-site assessment by the orthopaedic surgeon [27]. The waiting times of the referrals decreased on average from 201 to 40 days [27].

The authors concluded that by the use of telemedicine, it was possible to significantly reduce waiting list times and optimize travel times and expenditures [27]. In health-care crisis in which health-care resources are limited or restrictions for seeing patients in outpatient seeing are put in place, telemedicine might be a valuable option to protect health-care personnel against disease transmission and still provide sufficient service to a considerable number of orthopaedic patients. However, the authors also highlighted the fact that when using videoconferencing as a mode of consultation, the orthopaedic surgeon and traumatologist need to have specific physical examination skills as a manual examination is not possible [27].

During the current COVID-19 crisis, many orthopaedic surgeons have been forced to explore different methods other than face-to-face consultations, which is the conventional way for initial contact with the patient or clinical follow-ups. Video- or telephone consultations are increasingly recognized in these times due to regulatory restrictions, but also as many patients try to avoid showing up for a follow-up in hospital or the outpatient environment because they are afraid of nosocomial infections. It has also been shown that the use of telemedicine for the first consultation to an orthopaedics oncology service is highly cost-efficient, as it leads to a decrease in health-care cost between 12.2% and 72% [5].

## Discussion

The deaths of doctors during the COVID-19 pandemic may simply be a result of having been exposed in their work to people infected with the disease, whilst the general population has been in lockdown [4]. If this is borne out by the evidence, then sadly, this could have been avoided by providing full PPE as used in countries such as China and South Korea. Secondly, high viral load exposure has been linked with a more severe disease [6]. This may also have been avoided by providing full PPE for all patient contact, regardless of their infection status.

Orthopaedic and trauma surgery are the most common type of surgery in the emergency setting. The present literature review of the published evidence for PPE during orthopaedic surgery in a COVID-19 environment has highlighted the need for raised awareness of the types of PPE available for the surgical team, when they should be used and the definition of surgical AGPs. Interestingly, the WHO guidance on PPE for COVID-19 omitted the operating room, whereas UK NHS guidance evolved during the first few weeks of the UK lockdown [28, 33]. UK authorities eventually recognized surgery with high-speed devices as an AGP. Respiratory AGPs require FFP3 masks or powered air-purifying respirators, whereas surgical AGPs only require FFP2 masks [28]. Another option would be powered air-purifying respirators. Veterinary surgeons protect their surgical team from aerosilised Herpes virus particles using powered air-purifying respirators such as the 3 M Versaflo system with HEPA filter and an S533 cape [17].

It is widely accepted that COVID-19 virus is transmitted via droplets from the respiratory system of infected patients; however, the virus is also found in the blood and other bodily fluids. The clinical significance of non-respiratory tract viral transmission is unclear. The prevalence and importance of viral loads in the different fluids are widely unknown.

Bony surgery around the head and neck (ENT, neurosurgery and ophthalmic surgery) generates aerosols from the respiratory tract and exposes the surgeon to high viral levels. Orthopaedic surgery, particularly to the lower limb, produces vast amounts of aerosols including blood, fine bone particles, synovial fluid and saline from the irrigation when high-speed power tools are used (Table 1).

**Table 1:** Risk of procedures for aerosol generation (risk for AGPs): respiratory versus surgical aerosol

Surgery type	Level of surgical aerosol	Level of respiratory aerosol to the surgeon
ENT	High	High
Neurosurgery	High	Moderate (high for surgery at base of skull or trans-sphenoidal)
Ophthalmology	High	High
Orthopaedics	High	Low

The use of power tools is fundamental to osteotomies, joint arthroplasty and trauma surgery, where reaming, sawing and drilling are needed for bone preparation. Saline irrigation is necessary to reduce local tissue thermal damage, but can significantly add to aerosol generation.

Electrocautery generates smoke plumes, which contain bio-aerosols with viable and non-viable cellular material that subsequently poses a risk of infection (human immunodeficiency virus, hepatitis B virus, human papillomavirus) and causes irritation to the lungs [2]. COVID-19 is an RNA virus; however, transmission via this route is not known (Table 2).

**Table 2:** Types of surgical aerosol-generating techniques

Surgical technique	Level of surgical aerosol
High-speed power tools such as saws or burrs	High
Drill	High
Jet lavage systems	High
Electrocautery	High

**Fig. 1:**
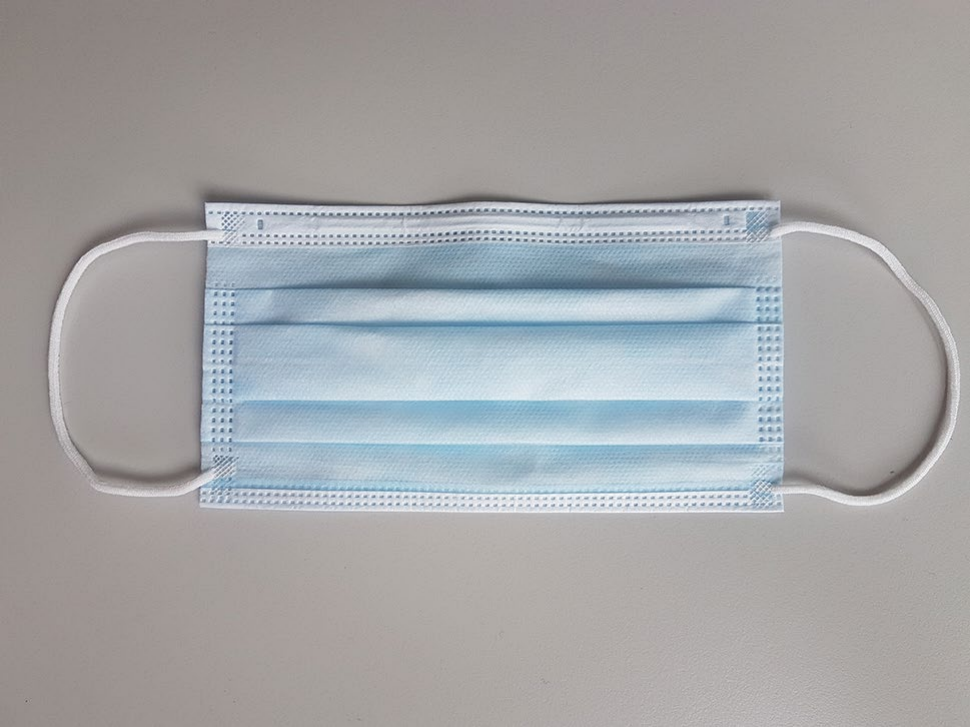
Single-use face mask

**Fig. 2:**
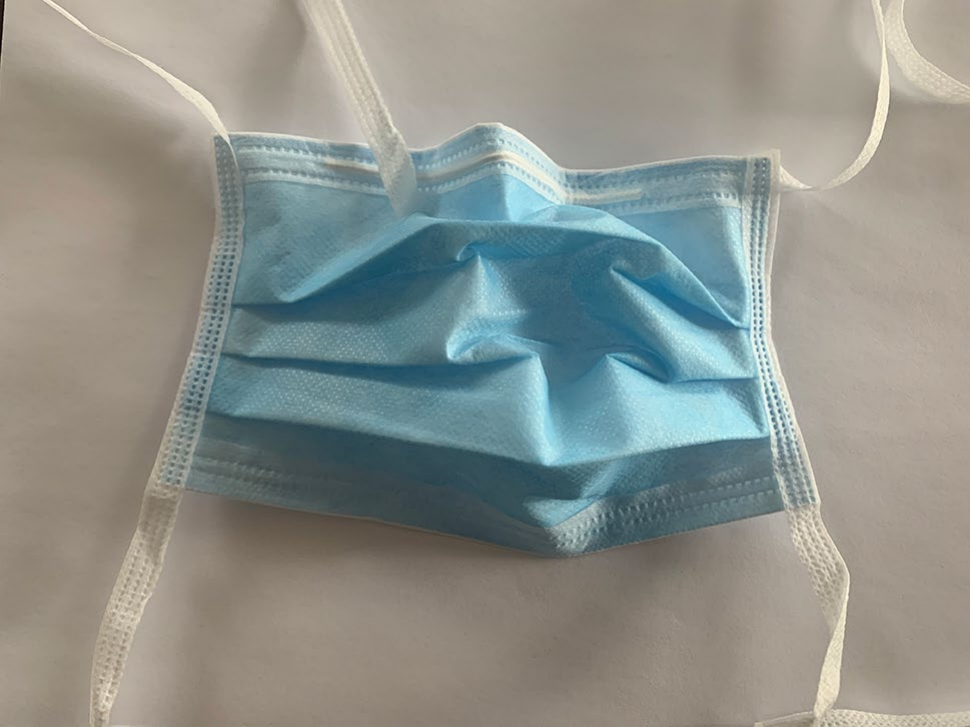
Surgical mask

European and US respiratory masks are classified into three protection classes. European respiratory masks and filtering face piece (FFP) are classified on their assigned protection factor and provide a level of protection to the user based on the concentration of the occupation exposure limit (OEL) (Figs. 1, 2).

FFP1 reduces OEL by a factor of 4, FFP2 by a factor of 10 and FFP3 by a factor of 20.

**Fig. 3:**
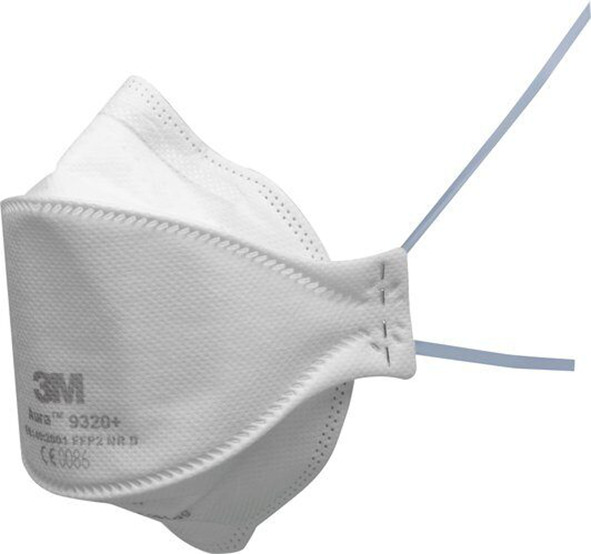
FFP2 mask

**Fig. 4:**
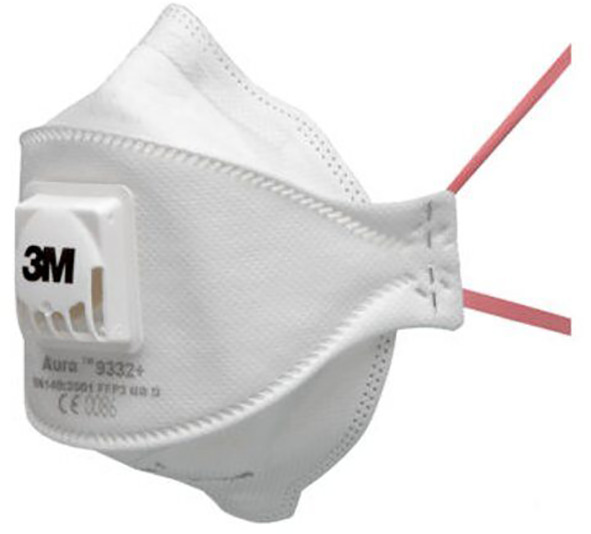
FFP3 mask

In the USA, the respiratory mask protection classification is based upon the percentage of filtration of very small particles (0.3 μm). They are classified into N95, N99 and N100 masks. The N95 mask blocks 95% of particles, the N99 blocks 99% and the N100 blocks 99.97% (Table 3). The European FFP1 does not offer protection against COVID-19. The European FFP3, equivalent to the US N99, is recommended for aerosol protection against COVID-19 (Figs. 3, 4).

**Table 3:** European and US standards for masks used for PPE

Filtration efficiency for particles of 0.3 μm	European standard (EN 149:2001)	US Standard (National Institute for Occupational Safety and Health (NIOSH))	Protection for health-care works against COVID-19
80%	FFP1	–	Not recommended No protection
95%	FFP2	N95	Good protection against airborne transmission
99%	FFP3	N99	Good protection against airborne transmission
99.97%	–	N100	Good protection against airborne transmission

With this review, it was found that different surgical power tools such as saws or burrs as well as electrocautery in cutting and coagulation mode lead to aerosol generation in the OR. According to the current knowledge, which is based on deductions from previous literature findings rather than specifically oriented research, aerosol generation puts the health-care workers at high risk for COVID-19 disease transmission. Hence, the recommended PPE for orthopaedic surgeons should consist of level 4 surgical gowns, face shields or googles and double gloves. In case of proven or suspected COVID-19 infection, orthopaedic surgeons should use FFP2-3 or N95-99 respirator masks (Table 4).

**Table 4:** Balanced recommendations for PPE in operating area for COVID-19-positive patients or suspected COVID-19 patients

	Health-care personnel (HCP)	Masks		Surgical gowns	Eye protection	Gloves
		Surgical	FFP1-3 N95-100			
Patient transport in and from OR	Persons involved in transport of patients	x	–	Level 1	–	x
Transfer of patient into OR area	All HCP	x	–	Level 1	x	x
Intubation and initiation of anaesthesia in OR	All HCP in OR	–	> FFP2/N95FFP3 N99	> Level 3	x When distance < 2 m	x
Surgery including surgical AGPs	All HCP in OR	–	> FFP2/N95FFP3 N99	> Level 3	x When distance	x (double glovin g)
	Occupational department personnel (ODP)	x	–	> Level 3	< 2 m x	x
Surgery including respiratory AGPs	All HCP in OR		> FFP2/N95FFP3 N99 or PAPR^a^ if surgeon needs it	> Level 3	x When distance < 2 m	x
	Occupational department personnel (ODP)	x	–	> Level 3	x	x
Extubation and ending of anaesthesia in OR	All HCP in OR	–	> FFP2/N95FFP3 N99	> Level 3	x When distance < 2 m	x
Cleaning of OR	Cleaning personnel	–	> FFP2/N95FFP3 N99	> Level 3	x	x

^a^Powered air-purifying respirator

x, indicated, –, not indicated

There is a number of limitations to be acknowledged with regard to the present review and PPE recommendations. There is currently ongoing change in evidence about PPE, hence this review is not meant to be a guideline; moreover, it aims to describe PPE in particular for orthopaedic surgeons and discuss the relevant clinical evidence. A systematic review was not possible due to the heterogeneity of available information.

## Conclusion

The COVID-19 crisis has alerted us to review current practice and evidence of personal protective equipment for orthopaedic and trauma surgeons. During orthopaedic and trauma procedures such as the use of power tools, burrs or electrocautery, potentially infective aerosol is generated. The major aim of our efforts should be to avoid an occupational transmission of COVID-19 by aerosolization of blood or other body fluids and hence adequate personal protective equipment should be available and used during surgery. In addition, efforts have to be made to improve the current evidence in this regard.
